# Decoding Stroke Etiology: Multi‐Omics Advancements in Genetic Mechanisms and Clinical Implication

**DOI:** 10.1002/brb3.70792

**Published:** 2025-09-01

**Authors:** Renxia Xiao, Mengxiao Zang, Xiaofeng Liu, Qingqing Zhu, Xiaojing Fu

**Affiliations:** ^1^ School of Nursing Shandong Second Medical University Weifang Shandong China; ^2^ Weifang People's Hospital Shandong Second Medical University Weifang Shandong China; ^3^ Weifang People's Hospital Weifang Shandong China; ^4^ School of Public Health Shandong Second Medical University Weifang Shandong China

## Abstract

**Purpose:**

Stroke has a high mortality and disability rate, yet its underlying mechanisms remain not fully understood. The occurrence of stroke is influenced by a combination of environmental factors, genetic factors, and their interaction, making its pathophysiology very intricate.

**Finding:**

With the development of genetic technology, researchers are endeavoring to explore the genetic risks of stroke from various perspectives, including genomics, transcriptomics, proteomics, and epigenetics, beyond common risk factors such as hypertension, diabetes, and heart disease.

**Conclusion:**

These studies have produced significant findings, identifying numerous risk genes, rare variants, copy number variations, and epigenetic characteristics associated with stroke, and enhancing our understanding of stroke occurrence, progression, and prognosis, and holding great promise for advancing personalized medicine with clinical stratification and risk prediction.

## Graphical abstract

This review systematically presents multi‐omics achievements in understanding the genetic susceptibility to stroke, including related risk genes, rare variations, CNVs, and epigenetic influences. Furthermore, it critically examines the clinical implications of these genetic discoveries for advancing personalized medicine, particularly in optimizing clinical stratification and enhancing risk prediction models.

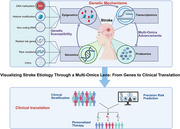

## 1 Introduction

Stroke, characterized by high disability and mortality rates, imposes a persistent and profound global health burden. Stroke includes ischemic (IS) and hemorrhagic strokes (HS), with ischemic stroke accounting for a significant proportion. Hypertension, diabetes, heart disease, obesity, and dyslipidemia are all identified risk factors for stroke (Potter et al. 2022). Despite advances, the pathophysiology of stroke remains complex and is not yet fully understood. Moreover, strokes with undetermined causes or lacking common risk factors are frequently observed.

Advancements in technology have accelerated the exploration of genetic risks related to stroke occurrence and progression. Researchers aim to elucidate the potential genetic susceptibility to stroke from multiple perspectives, including genomics, transcriptomics, proteomics, and epigenetics, to inform precision medicine.

This review aims to systematically describe “The Development and Clinical Application of Genetic Susceptibility in Exploring the Etiology of Stroke.” Based on the theme, a systematic literature search was conducted in the PubMed database using the keywords “stroke” and “genomics” as free‐text terms. The search was limited to English‐language articles published between January 1, 2020, and January 1, 2025. Citation tracking was employed as a supplementary strategy to identify relevant subsequent publications citing key foundational studies, ensuring comprehensive coverage of significant developments. Literature inclusion criteria: (1) Direct relevance to the review's theme; (2) Clear description of genetics/genomics‐related findings; (3) Priority given to studies with large sample sizes, studies integrating data from multiple databases, and studies encompassing diverse racial/ethnic populations. Exclusion criteria: Non‐English publications; animal studies; studies focusing solely on diagnosis or prognosis without addressing etiological mechanisms; conference abstracts; and commentary/editorial articles. The initial screening process identified 143 potentially relevant articles. Following a full‐text review and comparative analysis by the researchers, 75 high‐quality core publications were ultimately included in this systematic review.

Through systematic analysis, synthesis, and integration of the included literature, this review systematically presents achievements in understanding the genetic susceptibility to stroke, including related risk genes, rare variations, CNVs, and epigenetic influences. Furthermore, it critically examines the clinical implications of these genetic discoveries for advancing personalized medicine, particularly in optimizing clinical stratification and enhancing risk prediction models.

## 2 Progress of Genetic Factors Related to Stroke

### 2.1 Genomics and Stroke

Genomics has made remarkable progress in recent years, propelled by advancements in sequencing technology, genome editing, genome‐wide association studies (GWAS), and the integration of omics data. These advancements have revolutionized our understanding of biological systems and fostered their application in precision medicine. Specifically, genomics has profoundly impacted our understanding of stroke, providing insights into genetic risk factors, pathophysiological mechanisms, and potential for personalized medicine. These discoveries underscore the significance of individual pathways in stroke diagnosis and treatment. Researchers have uncovered key genes, genetic loci, and other biomarkers linked to stroke risk since the first ischemic stroke GWAS in 2007 (Matarín et al. 2007; Dichgans et al. 2021; Mishra et al. 2022). These genes and variations encompass various aspects, including vascular function, inflammatory response, and lipid metabolism (Gallego‐Fabrega et al. 2022; Williams et al. 2013; Palmisano et al. 2016; Jeanne and Gould 2017; Nielsen et al. 2018; Rozen et al. 2018; Farah et al. 2018).

The *HDAC9* gene is the first widely recognized causal gene for stroke (Malik et al. 2016). *HDAC9*, as a regulator of atherosclerotic plaque stability and IKK activation, especially increases the risk of large vessel stroke (Prestel et al. 2019; Asare et al. 2020). The PITX2 and ZFHX3 genes, which play roles in the development of the brain, heart, and blood vessels, have been found to affect an individual's stroke risk (Malik et al. 2016). In 2018, a multi‐ancestry genome‐wide association meta‐analysis of 521,612 individuals (67,162 cases and 454,450 controls) discovered 22 new stroke risk loci, bringing the total to 32 (Malik et al. 2018). Subsequently, multiple risk loci were identified, pinpointing the causal roles of specific genes and gene regions in stroke pathogenesis. Genetic variations, such as exonic polymorphisms in *NOS3*, *COL4A1*, and near *DYRK1A*, in the nitric oxide synthase‐nitric oxide pathway partially affect stroke risk through blood pressure variation (Malik et al. 2018). *ADAMTS12* has been identified as a new candidate gene for pediatric stroke (Witten et al. 2020). Variations in *DIAPH1* contribute to genetic susceptibility to stroke risk, particularly the small artery occlusion (SAO) subtype of IS (Ren et al. 2020). *CYP1A1* and *CYP1A2* variants are associated with stroke risk in the Chinese population (Mao et al. 2020). Three genes (SLC17A2, *FAM73A*, and OR52L1) and 32 loci, including 2 loci for cardioembolic stroke, 2 for small vessel disease stroke, and 4 for hemorrhagic stroke, were identified in an Indian population (Kumar et al. 2021). Nine new loci for the *CENPQ* and *ALDH2* genes were identified in a multiethnic GWAS meta‐analysis by The Global Biobank Meta‐Analysis Initiative (GBMI), a collaborative network of 23 biobanks from four continents (Zhou et al. 2022). One novel locus (13q33) and four novel loci for large artery stroke, as well as rare variants within the *C6orf26* gene suspicious for hemorrhagic stroke, were identified in another ancestrally diverse study from the Trans‐Omics for Precision Medicine (TOPMed) Program (Hu et al. 2022). The GIGASTROKE project, the largest multiethnic meta‐analysis GWAS in stroke to date (110,182 stroke patients from five ancestries and 1,503,898 controls), identified 89 loci (61 new) for stroke and stroke subtypes (Mishra et al. 2022). It is highly likely that, with the improvement and popularization of research methods, more valuable stroke‐related risk genes and genetic loci will be discovered.

In addition to common genetic variations, rare variants and copy number variations (CNVs) have gradually drawn attention in stroke genomics analysis. These variations may affect genetic stability and gene expression levels, thereby increasing stroke risk, either dependent on or independent of environmental influences. Malik et al. highlighted the important role of rare genetic variation, revealing that the presence of a rare variant in the *HTRA1* protease domain corresponded to a larger effect on WMH burden than meeting the criteria for hypertension (b = 0.26, SE = 0.02, *p* = 2.9×10^−59^) or being in the upper 99.8th percentile of a polygenic risk score distribution based on common genetic variants (b = 0.44, SE = 0.14, *p* = 0.002) (Malik et al. 2021). Xu et al. published an exome‐wide array analysis of 9721 stroke cases from the Stroke Genetics Network (the SiGN Network) and 12,345 controls, identifying 12 novel variants that were extremely rare in European Caucasians (MAF < 0.1%) but not in African American samples (Xu et al. 2022). Targeting the different prevalence of vascular risk factors (VRFs) in Chinese compared to Caucasians, 59 rare single‐nucleotide variants and insertions/deletions (InDels) were reported or predicted to be deleterious in genes related to VRFs and/or other stroke subtypes, and seven rare CNVs were found to be potentially related to intracranial atherosclerotic disease (ICAD) (Shi et al. 2022). In addition to common variants in *COL4A1/A2* related to sporadic intracerebral hemorrhage (ICH), two rare missense variants were also found to be associated with sporadic ICH (Chung et al. 2021). When rare variants of monogenetic diseases are analyzed in the general population, rare *NOTCH3* EGFR‐involving variants correspond to a genetic predisposition for age‐related cerebral small vessel disease and are identified as genetic risk factors for stroke and dementia in the general Chinese population (Wang et al. 2024; Liu et al. 2021). To further evaluate CNV‐associated stroke risk and outcomes, Cole et al. proposed a research project (2020–2025) named The Copy Number Variation and Stroke (CaNVAS), detailing research objectives, specific strategies, and feasibility (Cole et al. 2021). The results are worth looking forward to.

### 2.2 Transcriptomics / Multi‐Omics and Stroke

In exploring the genetic risks of stroke, the inclusion of transcriptomics and multi‐omics has significantly advanced our understanding. It not only bridges the gaps in the pathophysiological mechanisms linking genes and stroke but also facilitates the discovery of new stroke‐related genes and variations. The following represents a set of novel discoveries from transcriptomics and multi‐omics studies.

Traylor, M., et al. found that lacunar stroke had a substantial heritable component, except for a single locus on 16q24, by integrating GWAS and TWAS (transcriptome‐wide association study). This was associated with the expression of six genes (*SCL25A44*, *ULK4*, *CARF*, *FAM117B*, *ICA1L*, and TWASNBEAL1), implicating disruptions in the vascular extracellular matrix, phosphatidylinositol 5‐phosphate binding, and roundabout binding (Traylor et al. 2021). Polymorphism rs2953 of *CTNNB1* is reported to alter miRNA‐3161 binding, thereby affecting the risk of ischemic stroke (Zhao et al. 2021). Furthermore, integrative multi‐omics analysis (genomics, transcriptomics, and proteomics) is performed to deepen our understanding of the genes underlying stroke risk and their regulatory mechanisms. The GIGASTROKE project, the largest multiethnic meta‐analysis GWAS in stroke, utilized multi‐omics research approaches to reveal putative causal genes (such as *SH3PXD2A* and *FURIN*) and variants (such as at *GRK5* and *NOS3*) for stroke and its subtypes (Mishra et al. 2022). Another integrative multi‐omics analysis, based on multiple tissues, identified 136 susceptibility genes, splicing sites, eRNAs, and proteins spanning 60 (42 novel) genomic regions. It found that ischemic stroke (IS) risk was most enriched for expression quantitative trait loci (eQTLs) in arterial and brain‐related tissues (Jung et al. 2024).

### 2.3 Epigenetics and Stroke

Epigenetics refers to the study of heritable changes in gene expression or cellular phenotype that do not result from alterations in the DNA sequence (Bird 2007). These changes are frequently mediated through mechanisms such as DNA methylation, histone modification, and non‐coding RNA molecules, which critically regulate gene activity and maintain cellular identity. Epigenetic modifications are susceptible to environmental influences and have profound implications for development, disease, and inheritance (Kouzarides 2007; Goldberg et al. 2007).

Mounting evidence indicates that epigenetic mechanisms modulate cellular and molecular processes in both ischemic and hemorrhagic strokes (Huang et al. 2019; Shen et al. 2019; Hu et al. 2020; Zhu et al. 2021; Li et al. 2021; Wang et al. 2020). Kumar, A., et al. reviewed and concluded that epigenetic factors may serve as markers for disease progression, biomarkers for disease diagnosis, and targets for novel therapeutic interventions (Kumar et al. 2021). Morris‐Blanco et al. systematically reviewed the impact of epigenomics on stroke pathophysiology (Morris‐Blanco et al. 2022). The results showed that (1) blood levels of methylated DNA might serve as biomarkers for the diagnosis, prognosis, and treatment of stroke. (2) *HDAC9*‐mediated histone acetylation may contribute to genetic susceptibility to stroke, and polymorphisms in histone acetylation markers might mediate genetic risks in stroke. (3) While histone methylation marks are diverse and abundant, the role of histone methylation in stroke is still emerging. (4) Long noncoding RNAs function as epigenetic regulators in stroke, with their functional landscape still evolving.

A recent study shows that altered DNA methylation of the *TRIM6*, *TLN2*, and *FLRT2* genes may functionally contribute to atherosclerosis, thereby increasing the risk of ischemic stroke in Chinese populations (Peng et al. 2023). Moreover, an epigenome‐wide association study identified 20 novel loci associated with ischemic stroke (Soriano‐Tárraga et al. 2020). Regarding long noncoding RNAs, Zhang et al. summarize that circRNAs (circRNA HECTD1, circRNA DLGAP4, circRNA CDC14A, circRNA SCMH1, and circRNA TLK1) show promise as potential biomarkers for acute ischemic stroke (Zhang et al. 2023). Ahmed Elsabagh, D. T., et al. reported that long non‐coding ribonucleic acid growth arrest specific‐5 rs2067079 and micro‐ribonucleic acid‐137 rs1625579 may act as novel genetic markers for acute ischemic stroke risk (Ahmed Elsabagh et al. 2023). Akinyemi, R. O., et al. reported the potential roles of regulatory miRNA, intergenic non‐coding DNA, and intronic non‐coding RNA in the biology of ischemic stroke in individuals of African ancestry (Akinyemi et al. 2024).

## 3 Implications of Genetic Findings for Progress Towards Precision Medicine

### 3.1 Clinical Stratification

**FIGURE 1:**
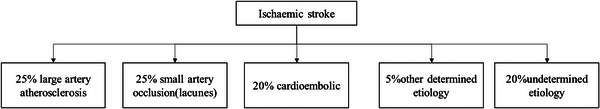
The TOAST classification (Adams et al. 1993, Hart et al. 2014).

The Trial of ORG 10172 in Acute Stroke Treatment (TOAST) classification constitutes a widely adopted international schema for the etiological typing of ischemic stroke (Adams et al. 1999). This schema categorizes ischemic strokes into five distinct types: large artery atherosclerosis, small artery occlusion, cardioembolic, stroke of other determined etiology, and stroke of undetermined etiology. The various subtypes exhibit differences in terms of etiology, risk factors, lesion distribution, and the severity of the condition, which in turn necessitates differential treatment focal points. HDAC9, as a key modulator of atherosclerotic plaque stability, exhibits a marked association with large artery atherosclerosis stroke (Prestel et al. 2019; Asare et al. 2020). Five loci (ICA1L‐WDR12‐CARF‐NBEAL1, ULK4, SPI1‐SLC39A13‐PSMC3‐RAPSN, ZCCHC14, and ZBTB14‐EPB41L3) were found to be associated with lacunar stroke, which is categorized as small artery occlusion stroke in TOAST classification (Traylor et al. 2021). DIAPH1 variants are linked to genetic stroke risk, particularly the SAO ischemic subtype (Ren et al. 2020). GP6 suggests a causal link with an increased risk of cardioembolic stroke in South Asian individuals (Wu et al. 2024). Incorporating polygenic risk for atrial fibrillation into clinical risk factors slightly enhances the differentiation between cardioembolic and noncardioembolic strokes and aids in the reclassification of stroke subtypes (Weng et al. 2023). ACE2 rs4646188 was associated with an increased risk of CS among diabetic patients in Uygurs (Weng et al. 2023). Polygenic risk scores (PRS) have value in identifying shared etiologies and determining stroke subtypes. Li et al. provided the first evidence that PRSs derived from MEGASTROKE have value in identifying shared etiologies and determining stroke subtypes (Li et al. 2021). Figure 1

In addition, genomics findings, particularly pharmacogenomics, can help customize treatment. The majority of stroke patients need to be treated with double antiplatelet agents in the acute phase, followed by long‐term secondary prevention with a single antiplatelet agent (Campbell and Khatri 2020; Bhatia et al. 2023). Unfortunately, the specific antiplatelet drugs do not have an antithrombotic effect in all patients, and they may be associated with a higher risk of adverse effects in certain patient populations (Al‐Shahi Salman and Greenberg 2023; Yip and Benavente 2011). Antiplatelet drug selection is critical in balancing efficacy and safety. The association of GP1BA, LTC45, PTGS1, and PTGS2 with aspirin use has been well documented. GP1BA has been demonstrated to have a substantial impact on the risk of aspirin resistance (Matsubara et al. 2008). The A allele of the PTGS1 gene has been shown to significantly increase the risk of aspirin resistance (Zhang et al. 2024; Li et al. 2024). PTGS2 is suggested as a potential genetic biomarker for individuals with ischemic conditions in Asia prior to aspirin therapy (Li et al. 2024). The C allele of LTC4S has been linked to aspirin‐induced urticaria (Mastalerz et al. 2006; Sánchez‐Borges et al. 2009). *CYP2C19*, a key enzyme in in vivo metabolism, influences the antiplatelet effect of clopidogrel (Holmes et al. 2011). Statins reduce the levels of low‐density lipoprotein cholesterol, stabilize atheromatous plaque, and are used as a first‐line therapy for stroke prevention (Campbell and Khatri 2020). Variations in the SLCO1B1 gene are associated with statin‐induced adverse reactions and reduced medication adherence (Kee et al. 2020; Voora et al. 2009). The 421C>A variant (rs2231142) in the ABCG2 gene causes a Q141K substitution, reducing the protein's export function in enterocytes and enterohepatic circulation. This leads to increased plasma statin concentrations and systemic exposure (Morisaki et al. 2005). CYP enzymes, particularly CYP2C9 and CYP3A4/5, significantly affect statin metabolism. Genetic variations in these enzymes can alter statin efficacy and safety. CYP2C9 polymorphisms notably impact fluvastatin pharmacokinetics, with certain genotypes leading to higher drug exposure and greater cholesterol reduction (Kirchheiner et al. 2003). For CYP3A4/5, genetic variations influence the metabolism of atorvastatin, simvastatin, and lovastatin (Willrich et al. 2009).

### 3.2 Risk Prediction

Beyond stratifying patients with stroke and guiding personalized medication, the achievements of genomics can also help assess and identify high‐risk populations for stroke, guiding primary prevention. Stroke is a well‐defined polygenic disease, and researchers have found that polygenic risk scores (PRS) or genetic risk scores (GRS) can identify and stratify subjects into different life‐long risk subgroups (Lu et al. 2021; Abraham et al. 2019). Integrating genetic risk assessment results with other factors can better guide the daily life and long‐term health care of high‐risk populations for stroke (Said et al. 2018; Rutten‐Jacobs et al. 2018).

The transition from unfavorable to favorable lifestyle practices significantly reduces the incidence of early‐onset cardiovascular diseases, particularly among individuals with high genetic risk. Specifically, in the high genetic risk group, such lifestyle improvements can reduce the risk of early‐onset coronary heart disease and ischemic stroke by 14.7‐fold. Unfavorable lifestyles in that research encompass smoking, poor dietary habits, lack of physical activity, unhealthy body weight, and large waist circumference (China Kadoorie Biobank Collaborative Group 2024). Hämmerle et al. reported that Genome‐Wide Polygenic Scores (PGS) capture distinct information about individual stroke risk, contrasting with family risk scores, which quantify an individual's risk of developing a specific disease (Hämmerle et al. 2022). A polygenic score integrating cross‐ancestry and ancestry‐specific stroke GWASs with vascular‐risk factor GWASs (integrative polygenic scores) strongly predicted ischemic stroke in populations of European, East Asian, and African ancestry (Mishra et al. 2022).

## 4 Conclusions

While substantial progress has been made in deciphering the genetic architecture and heritable risk factors underlying stroke, critical gaps persist in mapping the complete genomic landscape of cerebrovascular pathogenesis. The translational potential of these discoveries is particularly evident in their application to personalized stroke management, where genetic insights show promise for etiological classification of cryptogenic events, targeted prevention strategies, therapeutic target identification, and prognostic modeling. Nevertheless, persisting challenges in technology accessibility and cost‐effectiveness currently limit the broad implementation of genetic risk stratification in routine clinical practice. Four fundamental limitations constrain further progress: (1) pronounced geographic disparities in research infrastructure and implementation capacity, particularly across low‐resource settings; (2) inadequate representation of ancestral diversity in existing genomic databases, with current multi‐ethnic cohorts disproportionately focused on European‐ancestry populations; (3) insufficient integration of gene‐environment interactions and age‐related epigenetic modifications in risk prediction algorithms; (4) unresolved ethical dilemmas surrounding data privacy, incidental findings, and equitable access to emerging technologies. Addressing these challenges will require coordinated international efforts to establish equitable research partnerships, develop context‐specific implementation frameworks, and strengthen ethical governance in genomic medicine.

## Author Contributions

**Renxia Xiao**: writing–original draft, investigation, data curation, conceptualization, and methodology. **Mengxiao Zang**: data curation, investigation, and writing–original draft. **Xiaofeng Liu**: investigation and data curation. **Qingqing Zhu**: data curation and investigation. **Xiaojing Fu**: conceptualization, methodology, data curation, supervision, writing–review and editing, investigation, and project administration.

## Peer Review

The peer review history for this article is available at https://publons.com/publon/10.1002/brb3.70792

## Data Availability

Data sharing not applicable to this article as no datasets were generated or analyzed during the current study.
